# Using citizen science to describe the prevalence and distribution of tick bite and exposure to tick-borne diseases in the United States

**DOI:** 10.1371/journal.pone.0199644

**Published:** 2018-07-12

**Authors:** Nathan C. Nieto, W. Tanner Porter, Julie C. Wachara, Thomas J. Lowrey, Luke Martin, Peter J. Motyka, Daniel J. Salkeld

**Affiliations:** 1 Department of Biological Sciences, Northern Arizona University, Flagstaff, AZ, United States of America; 2 Department of Biology, Colorado State University, Fort Collins, CO, United States of America; University of Kentucky College of Medicine, UNITED STATES

## Abstract

Tick-borne pathogens are increasing their range and incidence in North America as a consequence of numerous factors including improvements in diagnostics and diagnosis, range expansion of primary vectors, changes in human behavior, and an increasing understanding of the diversity of species of pathogens that cause human disease. Public health agencies have access to human incidence data on notifiable diseases e.g., *Borrelia burgdorferi*, the causative agent of Lyme disease, and often local pathogen prevalence in vector populations. However, data on exposure to vectors and pathogens can be difficult to determine e.g., if disease does not occur.

We report on an investigation of exposure to ticks and tick-borne bacteria, conducted at a national scale, using citizen science participation. 16,080 ticks were submitted between January 2016 and August 2017, and screened for *B*. *burgdorferi*, *B*. *miyamotoi*, *Anaplasma phagocytophilum*, and *Babesia microti*. These data corroborate entomologic investigations of tick distributions in North America, but also identify patterns of local disease risk and tick contact with humans throughout the year in numerous species of ticks and associated pathogens.

## Introduction

Tick-borne pathogens have emerged or have expanded their geographic range throughout North America and are currently the most frequently reported vector-borne disease threat in the US [1–3]. For example, Lyme disease, caused by *Borrelia burgdorferi* sensu stricto and transmitted by *Ixodes scapularis* in the eastern US and *I*. *pacificus* in the western US, is the most abundant vector-borne disease in North America with an estimated incidence of >300,000 cases per year [1]. Additional pathogens transmitted to humans by these same tick species include the sister spirochetes *Borrelia miyamotoi* and *B*. *mayonii* which are newly recognized as human pathogens [4–7], as well as the causative agents of granulocytic anaplasmosis (*Anaplasma phagocytophilum*), human monocytic ehrlichiosis (*Ehrlichia chaffeensis*) and human babesiosis (*Babesia microti*) [3]. Other medically important hard-tick species include *Amblyomma* spp. and *Dermacentor* spp. that can transmit other *Ehrlichia* spp. and *Francisella tularensis* [8–11].

The incidence of tick-borne disease is the culmination of human exposure to an infected tick and consequent transmission of the pathogen. Data on the *incidence* of human disease rely on reports submitted to local, state and national health departments by laboratories, physicians and/or health care providers following diagnosis of illness in patients or identification of the pathogen in specimen samples [1]. Data on the *risk* of tick-borne diseases often depend on field-based observations of tick abundance and/or pathogen prevalence [12–13], but are often only relevant to a relatively small geographic area [14]. Unfortunately, descriptions of disease incidence and local risk do not actually describe patterns of exposure to tick bite i.e., where and when people come into contact with ticks. This type of data could be useful to reconcile information on local tick risk and incidence [15]. However, it is no trivial matter to understand rates of human contact, as researchers typically are limited by logistical and cost constraints to small-scale studies of tick-biting behavior [16].

Citizen science–when members of the public collaborate with scientists to collect data and samples–has recently been demonstrated as an effective technique to accomplish data collection and disease ecology surveillance at a scale unattainable by a limited research group of scientists alone [17–18]. For example, citizen science has been used to monitor the Asian tiger mosquito (*Aedes albopictus*) in Spain [19], and the spread of sudden oak death (caused by *Phytophthora ramorum*) in California [20]. Indeed, citizen science can offer information on national patterns in health issues and simultaneously convert the public from passive recipients of information to active participants in scientific study and, in effect, increase the body of scientific knowledge on an issue that is directly affecting them [17]. Citizen science approaches have previously been adopted to look at the ecology and epidemiology of tick-borne diseases in Canada, Finland and Massachusetts, US [18, 21–24]. Here, we report our first results on citizen scientist-collected data from across the US, to help broaden our geographic investigation and surveillance of ticks and tick-borne pathogens.

## Materials and methods

### Passive tick surveillance

From January 2016 until August 2017, Northern Arizona University offered a free tick identification and testing service made available to the general public i.e., citizen scientists, advertised through an initial public relation campaign and then made available to the public via a web site (Bay Area Lyme Foundation, http://www.bayarealyme.org/lyme-disease-prevention/tick-testing/). Broad geographic coverage was not systematically attempted however many Lyme Disease advocacy groups shared the website link on their own websites. Each submission included a description of an approximate location where the tick was encountered, habitat type, host type (human, pet, etc.), date encountered, and activity of the individual who encountered the tick. An email for a response was also included; however, no personal-identifying information was collected (e.g., name, address, gender). Importantly, submissions did not include information of recent travel history, and we accepted location information from the data submitted by the citizen scientist submitter without verification.

Ticks were sent to Northern Arizona University via mail and enclosed in zip-locked plastic bags. Upon arrival, ticks were identified to species, sex and stage using morphological characteristics [25–26]. For some species (e.g., *Dermacentor* spp.), using morphological traits to differentiate species can be difficult [27], but because the species are normally allopatric [28], we incorporated information on geographic range to allow species identification [29–30]. We did not evaluate engorgement status of the tick during identification. Individual ticks were then placed into 70% ethanol prior to nucleotide extraction.

### Pathogen detection

The program tested for the presence of *Borrelia burgdorferi*, *B*. *miyamotoi*, *Anaplasma phagocytophilum*, and *Babesia microti* via qPCR. Total DNA was extracted using the DNeasy blood and tissue kit (Qiagen Inc., Valencia, CA) following manufacturers recommendations with a few modifications. Ticks were bisected prior to placement in the Buffer ATL and Protease K, then incubated overnight at 56°C. In addition, during the elution step, 75 μl of sterile DI water was used as the elution buffer, columns were incubated at 56°C for 5 minutes prior to centrifugation, and the flow-through solution was then re-eluted for optimal DNA capture. The DNA solution was then placed into a freezer (-20° C) until further analysis.

All extractions were subjected to qPCR using previously developed primers and hybridization probes specific to each of the pathogens tested (Table 1). All assays were performed using SsoAdvanced Universal Probes Supermix 1X (Life Science Research, Bio-Rad, Hercules, CA) on a CFX96-TOUCH system (Life Science Research, Bio-Rad, Hercules, CA) and followed a two-step cycling protocol recommended by the manufacturer. Each 20 μl reaction contained primers at a concentration of 300 nM and probe at 250 nM (Applied Biosystems, Life Technologies, Carlsbad, CA) and included negative controls on every run. Samples were considered positive if they had a cycle threshold (C_T_) value < 40 and logarithmic amplification plots.

**Table 1:** Primer and probe sets used for the detection of tick-borne pathogens in ticks collected from throughout the US.

Pathogen	For Primer	Rev Primer	Probe	Reference
*Anaplasma phagocytophilum*	AGTTTGACTGGAACACACTGATC	CTCGTAACCAATCTCAAGCTCAAC	6FAM-TTAAGGACAACATGCTTGTAGCTATGGAAGGCA	[31]
*Babesia microti*	CAGGGAGGTAGTGACAAGAAATAACA	GGTTTAGATTCCCATCATTCCAAT	6FAM-TACAGGGCTTAAAGTCT-MGBNFQ	[32]
*Borrelia burgdorferi*	GCTGTAAACGATGCACACTTGGT	GCGGCACACTTAACACGTTAG	6FAM-TTCGGTACTA ACTTTTAGTTAA	[33]
*Borrelia miyamotoi*	GCTGTAAACGATGCACACTTGGT	GGCGGCACACTTAACACGTTAG	VIC-CGGTACTAAC CTTTCGAT TA	[33]

### Spatial and statistical analysis

County level-nationwide maps were created for each tick and pathogen species using ArcGIS 10.5 (ESRI, Redlands, CA). For each tick species, pathogen, and in each state we calculated the qPCR-prevalence and 95% confidence intervals by the proportions test (prop.test) in the statistical package “R” (R-Development Core Team, http://www.r-project.org).

## Results

### Tick submissions

**Fig 1:**
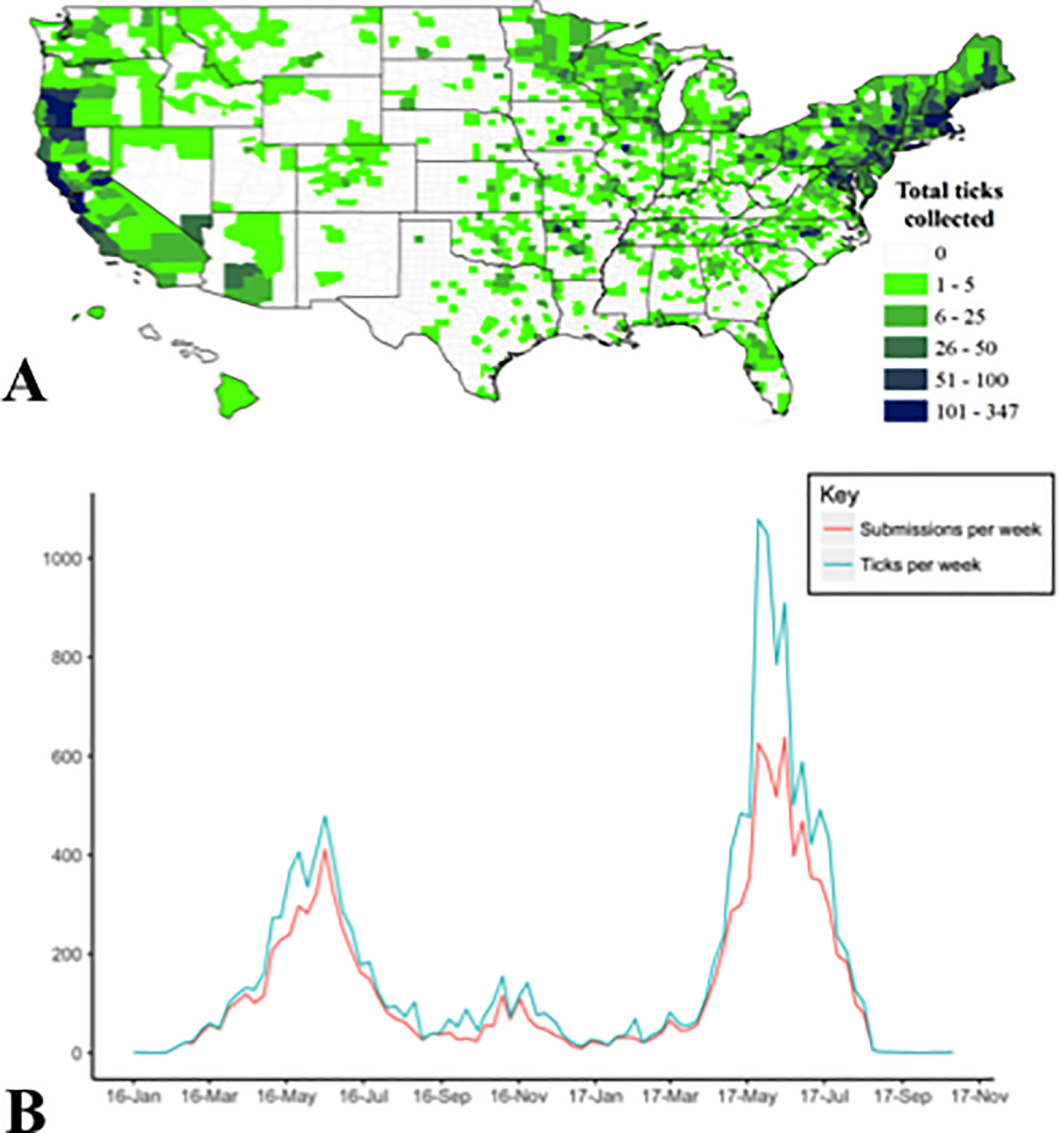
Submissions to the free tick testing program from across the US (A), starting in January 2017 ending in August of 2017 (B).

We received a total of 12,130 submissions from across the US’ 50 states and Puerto Rico, with an average of 1.33 (range = 1–84 ticks) ticks/submission, resulting in a total of 16,080 ticks collected and tested for pathogens (Fig 1A). Although 1.4% (223/16,080) of ticks were not morphologically identifiable due to decomposition or the removal of key morphological traits, these samples were still qPCR tested for pathogen presence. A total of 1.6% (265/12,130) of submissions were not ticks; these samples included insects (e.g. bees, beetles and ants), arachnids (e.g. mites), and scabs. None of the submissions from Alaska included ticks.

Ticks arrived in two annual pulses: one in the spring and a second smaller peak in the fall (Fig 1B). For example, in a single week in May 2017, we received ~600 submissions and ~1,000 ticks from across the US. The second year of surveillance, 2017, had a marked increase in submissions compared to 2016 e.g., 9,391 ticks collected in 7 months in 2017, compared to 6,532 ticks in 12 months in 2016.

Ticks were predominantly removed from human hosts (71.4%, 11,486/16,080) and dogs (17.1%, 2,746/16,080). Other host species included other domesticated animals (e.g. cats, rabbits), wildlife (e.g. deer, raccoons, rabbits) and livestock (e.g., goats, horses, pigs).

Submitted ticks were most often reported to have bitten their hosts (73.9%, 11,890/16,080), with the minority being reported as questing/crawling (23.4%, 3,766/16,080). Adult ticks comprised the highest number of submissions (80.0%, 12,867/16,080), followed by nymphs (17.7%, 2,846/16,080), larvae (0.9%, 144/16,080) and 223 (1.3%) unknowns (Table 2). Adult female ticks (68.2% 8,777/12,867 were submitted more frequently than male ticks (31.6%, 4062/12,867) and a small percentage (0.2%, 28/12,867) were not identifiable to sex.

**Table 2:** Summary of pathogens observed in ticks submitted by citizen scientists across the US [positives/sample size, (percentage prevalence, 95% CI)].

	*Anaplasma phagocytophilum*	*Babesia microti*	*Borrelia burgdorferi*	*Borrelia miyamotoi*
***Amblyomma***				
*A*. *americanum*	6/2078 (0.3, 0.1–0.7)	51/2078 (2.5, 1.9–3.2)	27/2078 (1.3, 0.9–1.9)	28/2078 (1.3, 0.9–2)
*A*. *maculatum*	1/ 21 (4.8, 0.2–25.9)	0/21 (0, 0–19.2)	1/21 (4.8, 0.2–25.9)	1/21 (4.8, 0.2–25.9)
*Amblyomma* spp. (Nymphs)	3/661 (0.5, 0.1–1.4)	9/661 (1.4, 0.7–2.7)	14/661 (2.1, 1.2–3.6)	9/661 (1.4, 0.7–2.7)
***Dermacentor***				
*D*. *andersoni*	1/132 (0.8, 0–4.8)	0/132 (0, 0–3.5)	0 / 132 (0, 0–3.5)	0/132 (0, 0–3.5)
*D*. *occidentalis*	1/264 (0.4, 0–2.4)	0/264 (0, 0–1.8)	2/264 (0.8, 0.1–3.0)	0/264 (0, 0–1.8)
*D*. *variabilis*	28/5853 (0.5, 0.3–0.7)	10/5853 (0.2, 0.1–0.3)	54/5853 (0.9, 0.7–1.2)	24/5853 (0.4, 0.3–0.6)
***Ixodes***				
*Ixodes pacificus*	23/2033 (1.1, 0.7–1.7)	0/2033 (0, 0–0.2)	67/2033 (3.3, 2.6–4.2)	36/2033 (1.8, 1.3–2.5)
*Ixodes scapularis*	191/4671 (4.1, 3.5–4.7)	86/4671 (1.8, 1.5–2.3)	765/4671 (16.4, 15.3–17.5)	60/4671 (1.3, 1–1.7)

### Geographic distribution of submitted tick specimens

We identified a total of 13 species of ticks from 49 states (all except Alaska) and Puerto Rico (Table 2, Fig 1A). The county-level distribution of each tick species generated by the citizen science submissions generally reflected the known distribution of each species, though there were some anomalies.

**Fig 2:**
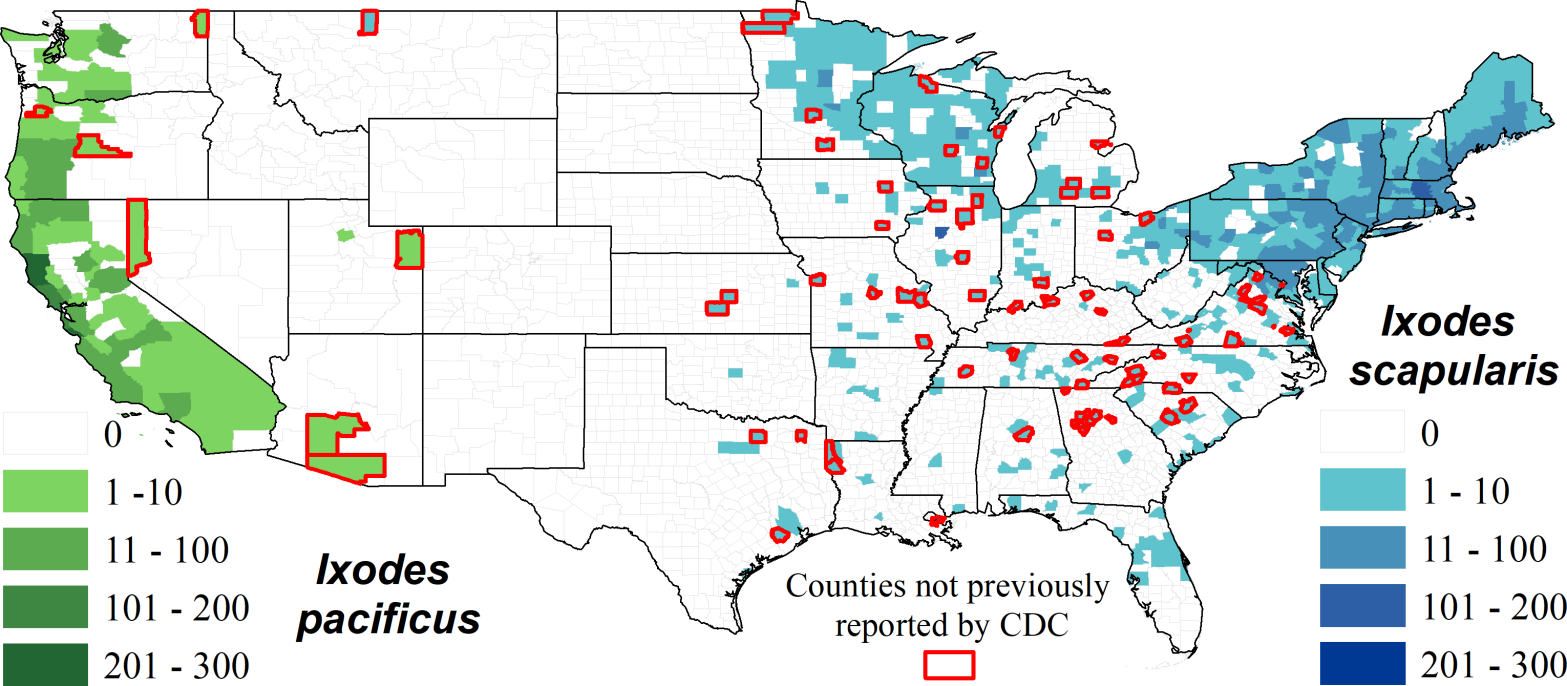
The county level distribution of *I*. *pacificus* and *I*. *scapularis* based on location data collected by citizen scientists. Counties outlined in red did not have previous records according to [12], no records include travel history of the submitter.

Both *Ixodes* species, *I*. *scapularis* (black-legged tick) and *I*. *pacificus* (western black-legged tick), were identified within their known distribution [12] though some new counties were identified as potential sources of these tick species. These ‘new’ county locations were most often adjacent or in close proximity to counties with an established presence of *Ixodes* ticks (Fig 2).

**Fig 3:**
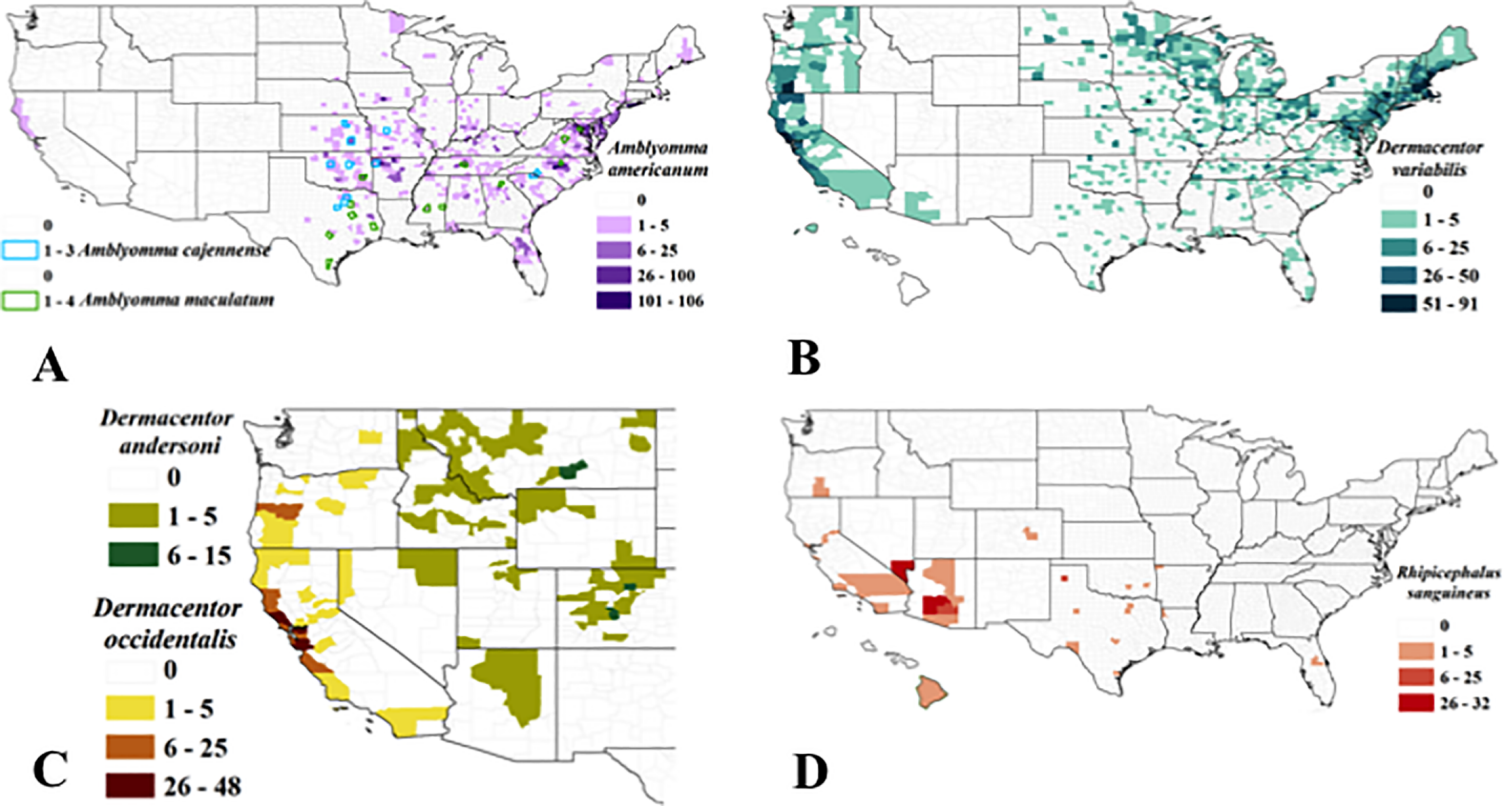
County level distribution of *Amblyomma* spp. (A), *Dermacentor variabilis* (B), *D*. *andersoni* and *D*. *occidentalis* (C), and *Rhipicephalus sanguineus* (D) identified following submission from citizen scientists.

The lone star tick, *Amblyomma americanum*, was identified throughout the eastern US, especially in the southeast, though also occasionally from individuals in far northern Wisconsin and Michigan, Maine and upstate New York. These incidents echo previous reported observations of the lone star tick [34]. We also received six *A*. *americanum* ticks from California (3 nymphs and 3 adults) (see Discussion). The Gulf Coast tick, *A*. *maculatum*, and the Cayenne tick, *A*. *cajennense*, were identified in southeastern US states, but in lower numbers than *A*. *americanum* (Fig 3A).

The American dog tick, *Dermacentor variabilis*, the Rocky Mountain wood tick, *D*. *andersoni*, and the Pacific Coast tick, *D*. *occidentalis* were submitted from counties corresponding to their current known distribution [25, 29–30, 35] (Fig 3B and 3C). One *D*. *variabilis* was also submitted from Puerto Rico.

We received brown dog ticks, *Rhipicephalus sanguineous* from a number of states in the southern US, western US, and from Hawaii (Fig 3D). Our pathogen screening did not detect infections in any of the *R*. *sanguineus* ticks submitted for testing (N = 119).

A few rare tick species were also submitted, including a single *I*. *spinipalpis* (from California), one *Haemaphysalis leporispalustris* (from Colorado), one *Otobius megini* (from California), and one *Ornithodoros hermsi* (from New Mexico), the rarity of which was expected given the high specificity of their life-history and tendency to feed on wildlife or domestic animal hosts [25] (Table 3). No pathogens were detected in any of these rare species.

**Table 3:** All the tick species collected as apart of citizen science collections separated by life-stage.

Tick Species	Total	Adult (%)	Nymph (%)	Larvae (%)
*Amblyomma americanum*	2078	1754 (84.4)	299 (14.4)	21 (1)
*A*. *cajennense*	14	14 (100)	0 (0)	0 (0)
*A*. *maculatum*	21	19 (90.5)	2 (9.5)	0 (0)
*Amblyomma* spp.	661	0 (0)	656 (99.2)	3 (0.5)
*Dermacentor andersoni*	132	132 (100)	0 (0)	0 (0)
*D*. *occidentalis*	264	263 (99.6)	1 (0.4)	0 (0)
*Dermacentor* spp.	7	2 (28.6)	5 (71.4)	0 (0)
*D*. *variabilis*	5853	5773 (98.6)	77 (1.3)	1 (0)
*Heamaphysalis leporispalustris*	1	0 (0)	0 (0)	1 (100)
*Ixodes pacificus*	2033	1785 (87.8)	225 (11.1)	17 (0.8)
*I*. *scapularis*	4671	2997 (64.2)	1560 (33.4)	98 (2.1)
*I*. *spinipalpis*	1	1 (100)	0 (0)	0 (0)
*Ornithodoros hermsi*	1	1 (100)	0 (0)	0 (0)
*Otobius megini*	1	0 (0)	0 (0)	0 (0)
*Rhipicephalus sanguineus*	119	112 (94.1)	7 (5.9)	0 (0)
Unidentifiable	223	14 (6.3)	14 (6.3)	3 (1.3)

### Tick-borne pathogen prevalence

A total of 16,080 ticks were tested individually for four tick-borne pathogens via qPCR (Table 4). All four pathogens were detected in *I*. *scapularis*, *D*. *variabilis*, and *A*. *americanum* submissions, the three species with the highest numbers of submissions (Table 2). While interesting, qPCR-positivity in the non-primary vector species may be more representative of exposure to pathogens, rather than the ability of the tick to competently transmit the pathogen.

**Table 4:** Prevalence of *A*. *phagotcytophilum*, *Bab*. *microti*, *B*. *burgdorferi*, and *B*. *miyamotoi* for *Ixodes scapularis* and *I*. *pacificus* ticks by state.

	n	*A*. *phagocytophilum*	*Bab*. *microti*	*B*. *burgdorferi*	*B*. *miyamotoi*
***Ixodes scapularis***					
Alabama	7	0 / 7 (0, 0–43.9)	1 / 7 (14.3, 0.8–58)	0 / 7 (0, 0–43.9)	0 / 7 (0, 0–43.9)
Arkansas	12	0 / 12 (0, 0–30.1)	1 / 12 (8.3, 0.4–40.2)	0 / 12 (0, 0–30.1)	0 / 12 (0, 0–30.1)
Connecticut	120	8 / 120 (6.7, 3.1–13.1)	6 / 120 (5, 2–11)	26 / 120 (21.7, 14.9–30.3)	1 / 120 (0.8, 0–5.2)
Delaware	10	0 / 10 (0, 0–34.5)	0 / 10 (0, 0–34.5)	1 / 10 (10, 0.5–45.9)	0 / 10 (0, 0–34.5)
Florida	25	0 / 25 (0, 0–16.6)	0 / 25 (0, 0–16.6)	1 / 25 (4, 0.2–22.3)	0 / 25 (0, 0–16.6)
Georgia	15	0 / 15 (0, 0–25.3)	0 / 15 (0, 0–25.3)	1 / 15 (6.7, 0.3–34)	0 / 15 (0, 0–25.3)
Illinois	149	8 / 149 (5.4, 2.5–10.7)	0 / 149 (0, 0–3.1)	9 / 149 (6, 3–11.5)	0 / 149 (0, 0–3.1)
Indiana	36	1 / 36 (2.8, 0.1–16.2)	2 / 36 (5.6, 1–20)	2 / 36 (5.6, 1–20)	0 / 36 (0, 0–12)
Iowa	13	0 / 13 (0, 0–28.3)	0 / 13 (0, 0–28.3)	1 / 13 (7.7, 0.4–37.9)	0 / 13 (0, 0–28.3)
Kansas	3	0 / 3 (0, 0–69)	0 / 3 (0, 0–69)	0 / 3 (0, 0–69)	0 / 3 (0, 0–69)
Kentucky	6	0 / 6 (0, 0–48.3)	0 / 6 (0, 0–48.3)	0 / 6 (0, 0–48.3)	0 / 6 (0, 0–48.3)
Louisiana	14	0 / 14 (0, 0–26.8)	0 / 14 (0, 0–26.8)	1 / 14 (7.1, 0.4–35.8)	0 / 14 (0, 0–26.8)
Maine	418	16 / 418 (3.8, 2.3–6.3)	7 / 418 (1.7, 0.7–3.6)	68 / 418 (16.3, 12.9–20.2)	1 / 418 (0.2, 0–1.5)
Maryland	230	1 / 230 (0.4, 0–2.8)	1 / 230 (0.4, 0–2.8)	13 / 230 (5.7, 3.2–9.7)	3 / 230 (1.3, 0.3–4.1)
Massachusetts	543	50 / 543 (9.2, 7–12)	14 / 543 (2.6, 1.5–4.4)	146 / 543 (26.9, 23.2–30.9)	9 / 543 (1.7, 0.8–3.2)
Michigan	31	0 / 31 (0, 0–13.7)	0 / 31 (0, 0–13.7)	2 / 31 (6.5, 1.1–22.8)	0 / 31 (0, 0–13.7)
Minnesota	126	5 / 126 (4, 1.5–9.5)	2 / 126 (1.6, 0.3–6.2)	16 / 126 (12.7, 7.7–20.1)	1 / 126 (0.8, 0–5)
Mississippi	4	0 / 4 (0, 0–60.4)	1 / 4 (25, 1.3–78.1)	0 / 4 (0, 0–60.4)	0 / 4 (0, 0–60.4)
Missouri	19	0 / 19 (0, 0–20.9)	0 / 19 (0, 0–20.9)	0 / 19 (0, 0–20.9)	0 / 19 (0, 0–20.9)
Montana	1	0 / 1 (0, 0–94.5)	0 / 1 (0, 0–94.5)	0 / 1 (0, 0–94.5)	0 / 1 (0, 0–94.5)
New Hampshire	173	12 / 173 (6.9, 3.8–12.1)	10 / 173 (5.8, 3–10.7)	43 / 173 (24.9, 18.8–32.1)	4 / 173 (2.3, 0.7–6.2)
New Jersey	185	6 / 185 (3.2, 1.3–7.3)	7 / 185 (3.8, 1.7–8)	29 / 185 (15.7, 10.9–21.9)	5 / 185 (2.7, 1–6.5)
New York	947	40 / 947 (4.2, 3.1–5.8)	23 / 947 (2.4, 1.6–3.7)	177 / 947 (18.7, 16.3–21.4)	10 / 947 (1.1, 0.5–2)
North Carolina	38	0 / 38 (0, 0–11.4)	0 / 38 (0, 0–11.4)	2 / 38 (5.3, 0.9–19.1)	1 / 38 (2.6, 0.1–15.4)
Ohio	182	3 / 182 (1.6, 0.4–5.1)	0 / 182 (0, 0–2.6)	12 / 182 (6.6, 3.6–11.5)	2 / 182 (1.1, 0.2–4.3)
Oklahoma	2	0 / 2 (0, 0–80.2)	0 / 2 (0, 0–80.2)	0 / 2 (0, 0–80.2)	0 / 2 (0, 0–80.2)
Pennsylvania	733	25 / 733 (3.4, 2.3–5.1)	2 / 733 (0.3, 0–1.1)	126 / 733 (17.2, 14.6–20.2)	17 / 733 (2.3, 1.4–3.8)
Rhode Island	36	1 / 36 (2.8, 0.1–16.2)	2 / 36 (5.6, 1–20)	11 / 36 (30.6, 16.9–48.3)	0 / 36 (0, 0–12)
South Carolina	18	0 / 18 (0, 0–21.9)	0 / 18 (0, 0–21.9)	0 / 18 (0, 0–21.9)	0 / 18 (0, 0–21.9)
Tennessee	30	1 / 30 (3.3, 0.2–19.1)	0 / 30 (0, 0–14.1)	0 / 30 (0, 0–14.1)	0 / 30 (0, 0–14.1)
Texas	11	0 / 11 (0, 0–32.1)	0 / 11 (0, 0–32.1)	2 / 11 (18.2, 3.2–52.2)	1 / 11 (9.1, 0.5–42.9)
Vermont	100	5 / 100 (5, 1.9–11.8)	0 / 100 (0, 0–4.6)	21 / 100 (21, 13.8–30.5)	0 / 100 (0, 0–4.6)
Virginia	203	2 / 203 (1, 0.2–3.9)	0 / 203 (0, 0–2.3)	14 / 203 (6.9, 4–11.5)	0 / 203 (0, 0–2.3)
West Virginia	29	2 / 29 (6.9, 1.2–24.2)	0 / 29 (0, 0–14.6)	2 / 29 (6.9, 1.2–24.2)	0 / 29 (0, 0–14.6)
Wisconsin	202	5 / 202 (2.5, 0.9–6)	7 / 202 (3.5, 1.5–7.3)	39 / 202 (19.3, 14.2–25.6)	5 / 202 (2.5, 0.9–6)
***Ixodes pacificus***					
Arizona	5	0 / 5 (0, 0–53.7)	0 / 5 (0, 0–53.7)	0 / 5 (0, 0–53.7)	0 / 5 (0, 0–53.7)
California	1649	18 / 1649 (1.1, 0.7–1.8)	0 / 1649 (0, 0–0.3)	51 / 1649 (3.1, 2.3–4.1)	23 / 1649 (1.4, 0.9–2.1)
Nevada	1	0 / 1 (0, 0–94.5)	0 / 1 (0, 0–94.5)	0 / 1 (0, 0–94.5)	0 / 1 (0, 0–94.5)
Oregon	308	3 / 308 (1, 0.3–3.1)	0 / 308 (0, 0–1.5)	14 / 308 (4.5, 2.6–7.7)	13 / 308 (4.2, 2.4–7.3)
Utah	2	0 / 2 (0, 0–80.2)	0 / 2 (0, 0–80.2)	0 / 2 (0, 0–80.2)	0 / 2 (0, 0–80.2)
Washington	69	2 / 69 (2.9, 0.5–11)	0 / 69 (0, 0–6.6)	2 / 69 (2.9, 0.5–11)	0 / 69 (0, 0–6.6)

#### Borrelia burgdorferi

*B*. *burgdorferi* qPCR-positive samples were predominantly detected in recognized primary tick vectors. In *I*. *scapularis*, the prevalence was 19.5% in adult ticks (585/2997, CI = 18.1–21.0), and 11.0% in nymphs (173/1560, CI = 9.6–12.8), and 5.1% in larvae (5/98, CI = 1.9–12.1) (overall prevalence of 16.4%, 765/4671, CI = 15.3–17.5). *I*. *scapularis* submitted from northeastern US states had the highest prevalence of *B*. *burgdorferi*, ranging from 6.5–23.1% (Table 2).

In contrast, in the western US, *B*. *burgdorferi* infections were highest in *I*. *pacificus* larvae with a prevalence of 5.9% (1/17, CI = 0.3–30.8), followed by 3.5% (62/1785, CI = 2.7–4.5) in adults and 1.8% (4/225, CI = 0.6–4.8) in nymphs (overall prevalence of 3.3%, 67/2027, CI = 2.6–4.2).

*B*. *burgdorferi* DNA was also identified in *A*. *americanum*, with the highest prevalence of 4.8% (1/21, CI = 0.2–25.9) in larval ticks, followed by 1.4% (24/1754, CI = 0.9–2.0) in adults and 0.7% (2/299, CI = 0.9–2.0) in nymphs (overall prevalence of 1.2% (27 / 2078, CI = 0.9–1.9). *Amblyomma* spp., nymphs and larvae (2.1%, 14/661, CI = 1.2–3.6) and an *A*. *maculatum* adult (5.2%, 1/21, CI = 0.3–28.1) tested positive for *B*. *burgdorferi*, as did *D*. *occidentalis* (0.8%, 2/264, CI = 0.1–3) and *D*. *variabilis* (0.9%, 54/5853, CI = 0.7–1.2).

#### Borrelia miyamotoi

The newly identified human pathogen, *B*. *miyamotoi*, exhibited a higher prevalence of infection in western black-legged ticks compared to the eastern congener. In the western US, prevalence was highest in larval *I*. *pacificus* ticks (5.9%, 1/17, CI = 0.3–30.8), followed by adults (1.8%, 33/1785, CI = 1.3–2.6), and nymphs (0.9%, 2/225, CI = 0.2–3.5), and an overall prevalence of 1.8% (N = 36/2027, 1.8%, CI = 1.3–2.5).

*B*. *miyamotoi* DNA was also identified in *I*. *scapularis* populations (1.3%, 60/467, CI = 1.0–1.7), with the highest prevalence in larvae (2.0%, 2/98, CI = 0.4–7.9), followed by adult ticks (1.4%, 41/2,997, CI = 1.0–1.9), and nymphs (1.1%, 17/1,560, CI = 0.7–1.8). Other tick species, not currently recognized as competent vectors, were also observed with *B*. *miyamotoi* infections: *A*. *americanum* (1.3%, 28/2078, CI = 0.9–2), an *A*. *maculatum* adult (4.8%, 1/21, CI = 0.2–25.9), *Amblyomma* spp. (1.4%, 9/661,CI = 0.7–2.7), and *D*. *variabilis* (0.4%, 24/5853, CI = 0.3–0.6).

#### Anaplasma phagocytophilum

*I*. *scapularis* samples contained the highest prevalence of *A*. *phagocytophilum* infections (4.1%, 191/4671, CI = 3.5–4.7) with adult ticks accounting for the highest prevalence (5.1%, 153/2,997, CI = 4.4–6.0), followed by nymphs (2.4%, 38/1560, CI = 1.8–3.4). *I*. *pacificus* also harbored *A*. *phagocytophilum* (1.1%, 23/2,033, CI = 0.7–1.7); prevalence was 1.2% in adult ticks (21/1,785, CI = 0.7–1.8) and 0.9% in nymphs (2/225, CI = 0.2–3.5). *A*. *phagocytophilum* DNA was also found in *A*. *americanum* adults (0.3%, 6/2078, CI = 0.1–0.7%), an *A*. *maculatum* adult (4.8%, 1/21, CI = 0.2–25.9), *Amblyomma* spp. (nymphs) (0.5%, 3/661 CI = 0.1–1.4). *D*. *variabilis* (0.5%, 28/5853, CI = 0.3–0.7%), *D*. *andersoni* (0.8%, 1/132 CI = 0–4.8), and *D*. *occidentalis* (0.4%, 1/264, 0–2.4) also had a low prevalence of *A*. *phagocytophilum*.

#### Babesia microti

*Amblyomma americanum* ticks contained the highest prevalence of *Bab*. *microti* (2.5%, 51/2078, CI = 2.5–3.2), with adults accounting for the highest prevalence of 2.6% (46/1754, CI = 1.9–3.5), followed by nymphs at 1.7% (5/299, CI = 0.6–4.0), and *Amblyomma* spp. nymphs (1.4%, 9/656, CI = 0.7–2.7). *Bab*. *microti* was also detected in *I*. *scapularis* (1.8%, 86/4671, CI = 1.5–2.3), with adults having a prevalence of 2.1% (64/2997, CI = 1.7–2.7) followed by 1.3% in nymphs (20/1,560, CI = 0.8–2.0). A low prevalence of *Bab*. *microti* was observed in *D*. *variabilis* (0.2%, 10/5,850, CI = 0.1–0.3).

#### Co-infections in ticks

Co-infections were identified in 0.98% (N = 158, CI = 0.8–1.2) of tested ticks, and *I*. *scapularis* accounted for 88.0% (139/158) of these co-infections. The most common co-infection was between *A*. *phagocytophilum* and *B*. *burgdorferi*, with an overall prevalence of 0.5% (83 / 16080, CI = 0.4–0.6%) and specifically in *I*. *scapularis*, 1.7% (79/4671, CI = 1.3–2.1). Co-infections with *Bab*. *microti* and *B*. *burgdorferi* also occurred with an overall prevalence of 0.2% (N = 36, CI = 0.2–0.3) and in *I*. *scapularis*, 0.7% (34/4,671, CI = 0.5–1.0). Co-infections were predominantly found in adult ticks (81.6%, 129/158, CI = 74.5–87.2). In addition, seven *I*. *scapularis* ticks were infected with triple infections of *B*. *burgdorferi-A*. *phagocytophilum-Bab*. *microti* (0.1%, 7/4671, CI = 0.07–0.3). No ticks were positive for all four pathogens.

## Discussion

### Citizen science surveillance

Our citizen science based collection method resulted in a relatively low cost and widespread collection of ticks from across the US, and provides insights into the patterns of tick exposure on humans, pets and in a few cases, wildlife. It is worth noting that this kind of science obviously taps into a desire for knowledge into disease ecology and epidemiology among the general populace. When the project was initiated, we expected approximately 2,400 submissions; and at times were almost overwhelmed by the actual 16,000-plus! Perhaps this response can be credited to the nature of the investigation i.e., examining human-wildlife interactions at the nexus of disease ecology and epidemiology is of an applied nature that is reflective of increasing concerns about disease emergence in society. Or perhaps the public interest was because the project facilitated tick-pathogen testing for free. Nonetheless, although the project did not make use of any concerted advertising campaign beyond the website and word-of-mouth (the program was easily accessible to all individuals seeking out tick testing via internet search engines, and was generally one of the first results to an internet search of “tick testing”), the submission of over 16,000 ticks demonstrates the potential to use citizen science as a surveillance tool for public health and vector-borne diseases.

We believe that utilizing the citizen science potential in a local area could be broadly effective at providing public health managers with a relatively cheap (reduced labor and travel costs) and effective (greater scope) way of determining the risk of particular tick species in a region. As mentioned earlier, this type of program has been used in other parts of the world and has helped to clarify the types of ticks and pathogens people come into contact with specifically. Additional information including travel history, knowledge of ticks and tick-borne disease in the area, and more specific demographic information about the citizen scientists would help us understand the biases involved in data collection.

Citizen science based sampling does have its limitations, including uneven awareness of the program and variation in the motivation of people who have ticks crawling or attached to them to actually submit the ticks as samples. These issues permeate passive surveillance programs generally. Concurrently, these data also have advantages in that they provide insights into geographical patterns or exposure to ticks and tick-borne pathogens that are not limited by jurisdictional boundaries, or by financial constraints and/or access to healthcare as the total cost for participants was simply the price required for mailing samples.

### Geographic distribution of tick species

Our data on geographic distributions of tick exposures corroborate and expand previously published records on tick distribution ranges. For example, we received *I*. *scapularis* from 594 counties and 35 US states and *I*. *pacificus* in 79 counties from 6 states. These data echo findings from an extensive literature search examining the reported presence of ticks within US counties: *I*. *scapularis* in 1,420 counties in 37 states, and *I*. *pacificus* in 111 counties and six states [12]. It therefore seems feasible that citizen science contributions can augment our scientific knowledge of where and when ticks are biting humans and animals, and perhaps can address our current inability to properly define current tick and pathogen geographic distributions, as well as to monitor changes in the ranges over time [12].

We did not collect travel history data of the people submitting ticks. Because ticks may remain attached, even on clothing, for several days our geographic distribution data for both tick and pathogen exposure must be regarded with caution [36–37]. However, ‘new’ county records are often not far from recognized tick or pathogen distributions and suggest either that prior efforts have not been adequate to report or collect ticks, the tick range is increasing, and/or that human and pet movement should be considered by physicians and/or health agencies when considering differential diagnoses for tick-borne diseases. A lack of data on travel history may also explain the small number of outliers were detected during the program e.g., the submission of lone star ticks (*A*. *americanum*) from northern California, and a single black-legged tick (*I*. *scapularis*) from northern Montana which are both locations well outside of the recorded range for these species. These anomalies could also reflect human error in identification, the potential import of the tick from either migratory wildlife or livestock to the area, or the fact that travel history was not included in the data [25, 38–39].

### Geographic distribution of tick-borne pathogens

For the most part, surveillance for pathogens also mirrored conventional perspectives on tick-borne diseases but our citizen-science based database also allows insights that are not available from the typical chains of information that rely on notifiable disease status by state. For example, babesiosis has been reported in 15/18 states where the disease is reportable, and predominantly in just seven states (97% of cases in just Connecticut, Massachusetts, Minnesota, New Jersey, New York, Rhode Island, and Wisconsin) [40]. We observed *Bab*. *microti* in several states where this pathogen is not a reportable disease (Table 3), and so although our data do not necessarily reflect an increase in geographical distribution, they do provide additional insight on the ecology and epidemiology of the disease. This kind of data could be used to inform predictive models of tick-borne disease spread that are currently restricted by the availability of human disease data [41]. Once again, this interpretation is subject to the caveat that we do not have travel data associated with the submitted ticks. In addition, the tick submissions confirm that babesiosis is a rare disease in the west coast; however the diversity of *Babesia* organisms in the west is likely unrealized (e.g., Washington, Oregon and California). For example, infection by *Bab*. *duncani* has occurred via transfusions in California, and *Bab*. *conradae* has been detected in dogs, however there is still no know vector or reservoir host for the piroplasms [42–43].

Nonetheless, citizen science-sourced data are not perfect. For example, our data did not observe *A*. *phagocytophilum* in Oklahoma, Arkansas or Missouri, where human cases have been identified [44]. Similarly, field surveillance efforts for ticks in California have demonstrated the presence of *B*. *miyamotoi* or *A*. *phagocytophilum* in counties that were not identified as locations for these pathogens based on citizen-science submitted ticks [45–46]. Obviously, when a pathogen is rare there will be more variation in the ability to detect the pathogen, which may be the case in the far western US. Additionally, our data identified a number of *B*. *burgdorferi* positive larvae which contradicts evidence the spirochete has limited, if any transovarial transmission. This however can be explained by the fact that the larvae tested come from poorly characterized sources and did not include engorgement status or infection status of the host. The result may be that the prevalence then that we estimate may be inflated.

Lyme disease transmission is associated with *I*. *pacificus* and *I*. *scapularis*, but we observed *B*. *burgdorferi* and *B*. *miyamotoi* in lone star, Gulf Coast, Pacific Coast and American dog ticks. However, these observations are presumably because these tick species feed on the reservoir hosts for these pathogens; these data do not on their own provide evidence that the tick species are involved in disease transmission. Indeed, xenodiagnostic experiments have failed to demonstrate viable pathogen transmission of *B*. *burgdorferi* by lone star or American dog ticks [47–49]. Previous studies have observed *Babesia* in lone star ticks, and suggested the potential for vector competence, but experimental studies are required to confirm pathogen transmission cycles in particular tick studies [49].

## Conclusions

In conclusion, citizen science data on tick ecology, combined with pathogen screening, offers insights into geographic patterns and distributions of these organisms at a scale that is difficult to compete with by laboratories or government agencies. These data can also be used at a more local scale to examine the phenology of human-tick encounters, geographical diversity of tick and pathogen genetics and so on.

## Supporting information

S1 Supporting Information County level data required to build the maps presented throughout the manuscript have been included as supporting information. (TXT)
